# How to manage with telemedicine people with neuromuscular diseases?

**DOI:** 10.1007/s10072-021-05396-8

**Published:** 2021-06-25

**Authors:** Emanuele Spina, Francesca Trojsi, Stefano Tozza, Aniello Iovino, Rosa Iodice, Carla Passaniti, Gianmarco Abbadessa, Simona Bonavita, Letizia Leocani, Gioacchino Tedeschi, Fiore Manganelli, Luigi Lavorgna, on behalf of Digital Technologies, Web and Social Media Study Group of the Italian Society of Neurology (SIN)

**Affiliations:** 1 Department of Neuroscience, Reproductive Science and Odontostomatology, Federico II University, Via Pansini, 5, 81028 Naples, Italy; 2 Department of Advanced Medical and Surgical Sciences, AOU University of Campania “Luigi Vanvitelli”, Caserta, Italy; 3 Experimental Neurophysiology Unit, Institute of Experimental Neurology (INSPE), San Raffaele Scientific Institute, Milan, Italy; 4 Vita-Salute San Raffaele University, Milan, Italy; 5 1st Clinic of Neurology, Italian Society of Neurology, AOU University of Campania “Luigi Vanvitelli”, Caserta, Italy

**Keywords:** Telemedicine, TeleRehabilitation, Tele-health, Neuromuscular disease, COVID-19

## Abstract

**Introduction:**

COVID-19 pandemic radically transformed our daily clinical practice, raising the need not to lose close contact with patients without being able to see them face-to-face. These issues are even more felt and evident in fragile patients, as those affected by neuromuscular disease. An important help came from new digital technologies that allow clinicians to remotely monitor health status and any deterioration of chronically ill patients.

**Methods:**

In this mini-review, an initiative of the “Digital Technologies, Web and Social Media Study Group” of the Italian Society of Neurology, we propose to analyze the approach to neuromuscular patients by looking over raising evidence on the main cornerstones of Telemedicine (TM): clinician-patient interaction, remote clinical assessment, remote monitoring, and digital therapeutics. In particular, we explored the strategies developed by researchers and their impact on the physical and emotional status of the patients, with particular focusing on their adherence to the program of virtual monitoring.

**Results:**

TM plays an important role in each of four stages of approach to neuromuscular disease, having demonstrated validity in keep close clinical patient interaction, clinical assessment, remote monitoring, and telerehabilitation. Nevertheless, there is no remote alternative to electrophysiological testing neither validate tools to assess disability.

**Conclusion:**

The role of TM in neuromuscular care is yet underestimated but is crucial, beyond the pandemic era. Further development of TM is advisable, through making specific apps, remotely controlled by clinicians, and making more engaging clinicians-patients interaction. Last, it is necessary to ensure adequate internet access to everyone.

## Introduction

The infection by the SARS-CoV-2 virus began in late 2019 in Wuhan, China, and soon spread all over the world, representing a healthy emergency for the entire world population. No country in the world has been spared from the infection, and considering its great diffusivity with an infection fatality ratio of around 1.3 % [1], healthcare systems have been highly put to the test.

Neuromuscular (NM) patients are considered “fragile patients,” at high risk to develop severe symptoms of COVID-19 infection, in particular patients affected by motor neuron disease, neuropathies with autonomic or respiratory muscle involvement, neuromuscular junction disorders, and dysimmune disease treated with immunosuppressive drugs [http://www.salute.gov.it/imgs/C_17_pubblicazioni_3014_allegato.pdf]. It is crucial in this class of patients to strictly adhere to WHO advise for preventing disease: maintaining social distancing, wearing correct face masks, avoiding crowded places and close contact, preferring outside meetings, and providing adequate hand hygiene [https://www.who.int/emergencies/diseases/novel-coronavirus-2019/advice-for-public].

On these indications, clinicians, and in particular neuromuscular specialists, tried to avoid exposing their patients to unnecessary outpatient visits. On the other hand, the health status of NM patients needs to be constantly assessed, to promptly catch any unexpected symptoms worsening and to avoid increasing anxiety or helpless feeling of these chronically ill patients.

In this frame, during 2020 and the beginning of 2021, telemedicine (TM) has experienced a huge development to maintain the provision of routine care in neurological and, more specifically, neuromuscular patients. Various approaches have been proposed for different diseases [2–7], but more in general, in the pandemic era, telemedicine provided the opportunity to maintain the patient-clinician alliance, reducing risks of outpatient evaluation for both patients and medical staff and becoming crucial in the management of neurological diseases resetting distances and pandemic obstacles.

As observed in other neurological conditions (e.g., parkinsonism, dementia, epilepsy, headache), TM has replaced in many cases outpatient visits [2–7]. Nevertheless, its use in NM care is yet dramatically underrated with few reports about the use of telemedicine care for neuromuscular diseases affected patients [8]. Here, we propose to review the approach to neuromuscular patients by analyzing raising evidence on the main cornerstones of TM: clinician-patient interaction, remote clinical assessment, remote monitoring, and digital therapeutics.

## Clinician-patient interaction

TM means “healing at a distance,” and, according to WHO definition, is “The delivery of health care services, where distance is a critical factor, by all health care professionals using information and communication technologies for the exchange of valid information for the diagnosis, treatment and prevention of disease and injuries, research and evaluation, and for the continuing education of health care providers, all in the interests of advancing the health of individuals and their communities,” and it is based on the following four elements:

1. Providing clinical support
2. Overcoming geographical barriers, connecting users who are not in the same physical location
3. Involving the use of various types of ICT
4. Improving health outcomes [https://www.who.int/goe/publications/goe_telemedicine_2010.pdf]

To do so, the first and most important feature of TM is to maintain close contact between clinicians and patients that, apart from the medical aspect, is crucial to provide psychological support to NM patients that are experiencing a dramatic social retirement with a sense of abandonment even on the part of the medical institution. Furthermore, TM could represent a simple tool to strengthen the doctor-patient alliance to facilitate the resumption of normal follow-ups at the end of the pandemic alert.

To facilitate clinicians-patients interactions, in the last year, various approaches have been proposed. In a little cohort (four siblings) of facioscapulohumeral dystrophy (FSHD), a team made up of neurologists, psychologists, pneumologists, and nurses, due to difficulties connected to reach referral center, try to apply a Telemedicine System based on systematic video-conferences and telemonitoring of cardiorespiratory variables. As result, the authors found a dropping in hospital admission and positive effects on mood; the very limit of this trial was the short duration, only 6 months [9]. Similar results were observed in three patients affected by various neuromuscular diseases, with a drop from 18 hospital admission in the period before TM management to 3 admissions during the TM period [10]. Moreover, underlying the importance of maintaining a strict contact in chronically ill patients, the greatest effects of TM on quality of life have been registered particularly on patients with moderate-to-severe disability, enlightening its key role in counteracting the sense of abandonment in fragile people [11].

Nevertheless, there is a lot to do under clinical aspect: a recent survey on remote consultation in Neurology daily practice showed the lowest grade of satisfaction among neuromuscular patients, probably due to feeling of needing direct clinical evaluation, and aged patients, probably due to their low familiarity with virtual devices [12]. Moreover, in many countries, authors could not ignore the issue raised by the inhomogeneous access to internet facilities of people affected by neuromuscular disease or technical issues of local institutions that have not yet provided to update their technologies.

## Remote clinical assessment

Moving from these bases, we should keep in mind that a peculiarity of neuromuscular evaluation is the request of a close clinician-patient contact to make a diagnosis. Moreover, as in the whole Neurology, clinical evaluation plays a key role in the approach to the patient: in Charcot-Marie-Tooth patients, for example, it is difficult to estimate a clinical worsening, since the patient’s health perception does not correlate with clinical disability [13, 14]. So how to overcome these limitations?

We must separate the neuromuscular approach into two steps: the neurological examination, including strength assessment, through evaluation of segmental strength, applying Medical Research Council (MRC, evaluating six pairs of muscles) [15]; sensory impairment evaluation (pinprick, touch, vibration sense) referred to every single nervous trunk; deep tendon reflexes (DTR) apart from classical neurological evaluation, from cranial nerves to cerebellar and gait assessment. Against the belief that in those patients an alternative to ambulatory visits is impossible, Saporta et al. recently proposed a “model” of tele-approach for neuromuscular patients. They replaced the MRC score for strength through assessment of specific muscle by video-performing of certain tasks; to acquire information about sensory systems, they instructed caregivers how to use a pin or cotton swab to test sensitivity; as regards the remaining neurological signs to examine and considered crucial for these patients (evaluation of balance and gait, the performance of cerebellar tasks), they are easy to evaluate through video assessment with an adequate caregivers training [15, 16]. Moreover, in specific conditions, like neuromuscular junction disease (e.g., Myasthenia Gravis), the neurological evaluation must include repetitive maneuvers to test fatigability that can easily be performed virtually [17]. Another interesting study proposed the introduction of the Veteran Affairs Neuropathy Scale (VANS), a remote examination tool useful to diagnose and monitor the severity of polyneuropathies with acceptable levels of sensitivity and specificity. Nevertheless, this scale needs real validation, beyond pilot study [18].

Another key feature in neuromuscular care evaluation is the assessment of the overall disability: changes in patients’ disability may be caught through easy-to-administer tools (in most cases, NM patients are cognitively intact), useful to assess functional autonomy in many NM diseases, as the Overall Neuropathy Limitations Scale (ONLS) [19] or the inflammatory Rasch-built Overall Disability Scale (i-RODS) [20]. ONLS measures the grade of disability for upper and lower limbs separately by assessing activities of daily life limited by the neurological impairment (e.g., washing hair, turning key in a lock, doing button or zips, walking with or without aid), while i-RODS consists of questionnaires about limitations in every-day life tasks (from reading magazines to doing dishes or dancing) and is used mainly to identify gross changes in health status. The changing in these scorers helps the clinician to quantify the disease worsening to address a cycle of pharmacological or physical therapies. Both these scales could be easily performed by phone interview or during telematic consult in few minutes.

However, except i-RODS scale [20], there is no validation of these approaches in routine care; nevertheless, considering the necessity to develop remote surveillance regardless of the pandemic era (e.g., patients with reduced mobility), the evolution of care in Neurology and neuromuscular care must pass from the use of alternative methods with respect to classic outpatient visits that had to remain of primary importance but to be reserved only when strictly appropriate.

Regarding clinical assessment, a very important contribution was given by a recently published analysis on 2999 telemedicine encounters in which, comparing the effectiveness of visit for each neurology sub-specialties, authors concluded against the perception that sensorimotor system examination in TM visits is more challenging in neuromuscular care, not having observed any difference in safety and quality outcomes respect to other diagnoses, as epilepsy [21].

The true limitation of a telemedicine application in neuromuscular care, which could not be addressed in an alternative way, is electrodiagnostic testing (EDX), which provides complete information on nerves, muscles, and nerve-muscle interface status. The EDX has a wide spectrum of use in NM cares, and is crucial to make the diagnosis and to monitor any deterioration and cannot be surrogated through tele-assessment [22].

Indeed, few urgencies require prompt execution of EDX testing. According to a recent review [23], clear clinical pictures that need urgent EDX are Guillain-Barrè syndrome and myasthenic syndrome in which delays in obtaining antibody titers could be detrimental for patients; in other cases, as suggested also by Italian neurophysiologists [24], EDX testing should be postponed, except fast progressive cases of inflammatory neuropathies/myopathies or clinically unclear amyotrophic lateral sclerosis [23]. Furthermore, conditions that require urgency in performing EDX testing are frequently life-threatening, with the need for in-hospital management; so there is no necessity of alternative assessment as in chronic management, which remains the main target of telemedicine in neuromuscular care. Otherwise, we can suggest requesting polymerase chain reaction studies on nasopharyngeal swabs 24h in advance of the EDX study that cannot be postponed anymore.

## Remote monitoring

TM has a huge potential to explore. Apart from the role of replacing ambulatory evaluations, other possible applications of telemedicine are in the development of tele-health applications to remotely control in a continuous way patients. By using devices or apps (as it already happens in other neurological diseases like multiple sclerosis) [25], possibly connected to the referring tertiary neuromuscular center, patients can provide clinicians daily information about their health status. This information appears to be useful to monitor not only general condition (e.g., blood pressure, cardiac rhythm, oxygen saturation), but also ambulatory performances (e.g., walking speed, balance, or resistance performance, as in the 6-min walk test, recently proved as a reliable tool to evaluate patients health status changes) [26], the entity of body movements (actigraphs or accelerometers) and strength (vigorimeter), letting clinicians able to monitor by remote patients' health status and to decide whether to program or delay new virtual or face-to-face clinical assessment. As regards general condition monitoring, in the yet cited study on four FSHD siblings, Telemedicine System provided three times a day cardiopulmonary parameters to the Control Center in case of stable disease; otherwise in case of the unstable condition, it provided 24-h 7-day information on patient health status to skilled operators at Control Centers directly in contact with a specialist in case of urgent needs [9]. However, main advances in remote monitoring have been conducted in ALS patients, in which remotely connected devices for monitoring pulmonary performance have been introduced for 10 years [27]. Regarding specific concerns in neuromuscular care, TM should include remote monitoring of muscle strength, actigraphy, and electrical impedance myography with daily transmission to the referral center, as underlined in a recent opinion on optimization of remotely conduct clinical trials in ALS (Table 1) [30]. A recent study on portable activity monitors for idiopathic inflammatory myopathies measuring average step counts/minute; peak 1-min cadence showed high compliance of patients in wearing devices (revealing a high level of patients commitment toward disease) and a very high level of concordance of these measurements with classical in-patient or ambulatory clinical evaluation. It demonstrated validity in register changes in the physical status of neuromuscular patients, with early recognition of worsening [28].

**Table 1:** Adherence and efficacy in TM clinical trial for neuromuscular care

Authors	Disease	Design	Tool	Adherence	Outcome and efficacy	Strength	Limits
Portaro et al., 2018 [9]	FSHD	Open label, only treated group	Video-conferencing, telemonitoring of cardiorespiratory variables and telerehabilitation	All patients completed the study	Dropping of hospital admission for check-up; mild improvement in mood and emotional status of all the patients	Increased autonomy and self-monitoring preventing unnecessary hospital admission	Short duration (only 6 mo)
Zamarron et al., 2013 [10]	FSHD, ALS, DMD	Open label, only treated group	Remote monitoring of biological signals, video-conference	All patients completed the study	Acceptable level of satisfaction, dropping of hospital admission (18 to 3 in a previous period of same duration)	Long-term monitoring (5 yr); high level of feasibility	No existing standard of care in telemedicine; lag in video connections,
Martinez et al., 2021 [11]	Rare neuromuscular disease	Open, treated vs control	Tele-assistance via skype video-conferencing in 7 wk	All patients completed the study	Improving of quality of life in patients with moderate (both physical and psychosocial field) to severe (only psychosocial) disability degree	Design of the study, sample size, multidomain evaluation	technical problems; outcome measures not specific for diseases
Rockette-Wagner et al., 2021 [28]	Inflammatory myopathy	Open label, observational	Remote monitoring with wearable devices, FDA approved	29/44 (66%) provided valid data at baseline and follow up	Portable devices output variables had good reliability and validity for changes in the physical activity of IIM patients	Large patients cohort and longitudinal data	Output variables of portable devices not yet validated; cohort heterogeneity,
Sobieranska et al., 2020 [29]	DMD	Open label, observational	Tele rehabilitation with online workshop	45 respondent out of 69 enrolled (69%), 16 (23%) participated in live workshop; 132 total visualization of online videos	Online videos are more acceptable than live sessions; motor assessment results will be presented	Limited data available	Limited data available

## Digital therapeutics

There are many different neuromuscular diseases with very different treatments. As regards pharmacological approaches, we can divide them into in-hospital and out-hospital treatments. Some in-hospital treatments should not be postponed or surrogated, as antisense oligonucleotide (ASO) treatment in spinal muscular atrophy (SMA), inotersen and patisiran administration for transthyretin amyloidosis (ATTRv), ASO for muscular dystrophy of Duchenne/Becker (DMD/BMD), glucosidase alfa for Pompe disease, or rituximab in chronic inflammatory demyelinating polyneuropathy (CIDP) [31]. Nevertheless, some in-hospital treatments can be replaced: for example, in chronically affected dysimmune patients who need monthly IVIG, as it happens in CIDP, the clinician can switch to home-administered subcutaneous immunoglobulin formulation, with remote-video strict control of the disease course. Regarding home therapies, long-term oral steroids or immunosuppressive agents (azathioprine, methotrexate) should be continued without interruption [32, 33]. According to this approach, the survey of the Italian Association of Myology demonstrated a satisfactory level of neuromuscular care only in continuing pharmacological therapies, mostly thanks to chronic home therapy (93.3% of the pre-COVID level) [34].

The same survey depicted instead a tragic picture on a physical therapy session with a 93% of reduction; physical therapy is essential in the management of those patients, which in most cases are chronically affected and need continuous physical stimulation to optimize their residual function [34]. Most of this reduction is due to the closure of hospital clinics of rehabilitation and limiting home rehabilitation, raising a big issue in the daily management of neuromuscular patients and imposing the need to find an alternative approach for these patients because according to the guidelines of the Italian Society of Physical Medicine [http://www.simferweb.net/varie_sito_simfer_allegati/varie/lineeGuida/LINEE-GUIDA_PER_LA_RIABILITAZIONE_DELLE_MALATTIE_NEUROMUSCOLARI_INFANTILI_DI_ORIGINE_GENETICA/2003-09-08_Mal_NMgenet.pdf] neuromuscular diseases need daily physical treatment. If for stroke and multiple sclerosis the role of telerehabilitation had been widely assessed [https://snlg.iss.it/wp-content/uploads/2018/05/Santilli_Vol_LineeGuida_MFR_parte-2_bis.pdf], there is no mention in the literature of telerehabilitation in NM diseases, except one recently published paper, proposing a model of telerehabilitation in DMD patients. In that study, the authors established an online program of rehabilitation based on virtual workshops, videos with exercise instructions with the participation of multidisciplinary team, and/or individual online consultations. The possibility to continue online their rehabilitation was positively received by patients and caregivers, with high level of usage [29]. By enhancing the engagement of NM patients and by developing a specific model of telerehabilitation for NM care, the goal is to improve the physical and mental health of our patients, decreasing trips to the hospital and amount of pharmacological therapies.

## Conclusion

Concluding, COVID-19 had dramatically changed our way to manage healthcare, introducing new techniques to follow up and visit patients. The role of telemedicine in neuromuscular care is yet underestimated but will be crucial, beyond the pandemic era, in avoiding useless evaluation in chronically ill patients, in managing routine care, and in identifying those patients who need urgent and direct clinical or electrophysiological assessment. Further development of TM is advisable, through making of specific apps, remotely controlled by clinicians, useful to monitor daily changes in physical performances of neuromuscular patients and through ensuring adequate internet access to everyone.
